# Intentions to leave and associated factors among laboratory professionals working at Amhara National Regional State public hospitals, Ethiopia: an institution-based cross-sectional study

**DOI:** 10.1186/s13104-019-4688-z

**Published:** 2019-10-14

**Authors:** Endalkachew Dellie, Gashaw Andargie Biks, Geta Asrade, Tsegaye Gebremedhin

**Affiliations:** Department of Health Systems and Policy, Institute of Public Health, College of Medicine and Health Sciences, University of Gondar, PO. Box: 196, Gondar, Ethiopia

**Keywords:** Intention to leave, Laboratory professionals, Public hospitals, Amhara region, Ethiopia

## Abstract

**Objective:**

Laboratory professionals play a vital role in the detection, diagnosis, and treatment of diseases. Knowledge of workplace variables that either motivates staff to keep working or quit their jobs is important for decision making. Thus, this study aimed to assess intentions to leave workplace and associated factors among laboratory professionals working at public hospitals of the Amhara National Regional State, Ethiopia.

**Results:**

An institution-based cross-sectional study was conducted from February 16 to March 14, 2016, among 336 randomly selected laboratory professionals. The study revealed that 65.5% (95% CI 60–70) of professionals had intentions to leave their hospitals. Dissatisfaction with the provision of educational opportunities (AOR: 3.59, 95% CI 1.61–7.99), poor pays and benefits (AOR: 3.89, 95% CI 1.53–9.89), lack of recognition (AOR: 2.69, 95% CI 1.35–5.38), poor working environments (AOR: 2.77, 95% CI 1.45–3.30), high workload (AOR: 1.94, 95% CI 1.04–3.63), low affective commitment (AOR: 2.05, 95% CI 1.10–3.82), and being unmarried (AOR: 2.46, 95% CI 1.32–4.58) were factors significantly associated with intentions to leave. Magnitude of laboratory professionals’ intention to leave was so high. Healthcare policymakers and hospital managers need to develop and institutionalize evidence-based retention strategies to reduce the intention of laboratory professionals to leave their workplace.

## Introduction

Laboratory professionals (LP) play a vital role in the detection, diagnosis, and treatment of diseases. Although they have been among the most neglected cadres in the health systems of sub-Saharan Africa. They often work in poorly equipped facilities which do not adhere to safety and infection control standards [1, 2]. Thus, laboratory staff get dissatisfied and lose faith in their profession. As a result, high staff turnover has been affecting the performance of laboratory professionals’ and ultimately, the quality of clinical care [3, 4].

The intention to leave is a subjective individual prediction about leaving jobs of the moment, working places or organizations in the near future, which is considered as a proxy of actual leaving [5, 6]. According to studies on the issue, several factors contribute to intentions to leave. Job satisfaction (pays and benefits, work autonomy, coworker relationships, supervisions, working environments, and working conditions) and organizational commitment are the most important factors which play an important role in determining employee intentions to leave jobs or their organizations [7].

Health professionals’ intention to leave significantly affects the functioning of the health care sector worldwide, especially in developing countries, and it impedes progress towards different health-related goals [8, 9]. It affects organizations’ capacity to achieve objectives by reducing innovation, affecting the quality of services and the motivation of employees. Besides, it is very costly for organizations [9, 10].

Employee intentions to leave and instability at health facilities are high, particularly in developing countries [11, 12]. In Southern Ethiopia, 59.4% of the health professionals intended to leave their workplaces [13]. Similarly, in the Amhara region, 60.2% of the nurses reported they wanted to leave their current working places [14].

The relationship between intentions to leave and other variables were uncertain in most research reports. Considerable research focused on the shortage and intentions to leave workplaces in the field of nursing [15]. However, staffing shortages and turnover intentions are affecting other health care professions as well. The reasons behind the intensions of LPs to leave workplace however remain unknown. Therefore, this study set out to address workplace variables that influence LPs’ intentions to leave their workplaces.

## Main text

### Methods

#### Study design and setting

An institution-based cross-sectional study was conducted in the Amhara National Regional State (ANRS) public hospitals from February 16 to March 14, 2016. Located in northwest Ethiopia, the regional state has 40 functional hospitals (5 referral, 3 general, and 32 primary) and 834 health centers with a total of l,699 laboratory professionals [16]. The study population was all LPs in ANRS public hospitals who had worked for at least 6 months before the study.

#### Sample size and sampling procedures

The sample size was determined using 52.5% proportion of health professionals’ turnover intentions [17] by considering a population correction formula, 1.5 design effect, and 10% non-response rate, which yielded the final sample size of 366. Study participants were selected using a stratified cluster sampling technique. LPs were stratified based on the level of working organizations (primary, general, and referral hospitals). Then, the calculated sample size was proportionally allocated to each stratum. Three referral, two general, and sixteen primary hospitals were selected from each stratum by using the lottery method. Finally, all laboratory professionals at each selected hospital were included in the study.

#### Measurements

Data were collected using a structured self-administered questionnaire first prepared in English and translated into the local language (Amharic) and retranslated to English to maintain consistency. The dependent variable, intention to leave the workplace was measured by three items following the Mobley et al. definition [18]. Respondents were asked to indicate the extent of their agreement using a five-point Likert scale (1: strongly disagree to 5: strongly agree). Respondents who scored more than 60% of the sum of all the intention to leave scale items were considered as showing the intention to leave [19].

Job satisfaction was measured by a five-point Likert scale of 72 items that included 12 subscales like (pay and benefit (5 items), supervisor support (12 items), policy and strategy (5 items), coworker relationship (5 items), training opportunity (6 items), the nature of work (10 items), responsibility (3 items), autonomy (3 items), workload (7 items), performance appraisal (3 items), recognition and reward (4 items) and work environment (7 items). Laboratory professionals who scored > 60% of the sum of the satisfaction scales were considered as satisfied [19]. The reliability of the tool for each subscale was checked using Cronbach’s alpha reliability test with a score of greater than 0.78.

Organizational commitment (affective, normative, and continuance commitment) was assessed using the scales developed by Meyer and Allen [20]. A five-point Likert scale (1: strongly disagree to 5: strongly agree) of three items for each component were used. A score with more than 60% of the sum of the commitment scales represented a high organizational commitment.

#### Data processing and analysis

The completed data were entered into Epi-info Version 7 and exported to SPSS (Statistical Package for the Social Sciences) version 20 software for analysis. Both bi-variable and multi-variable logistic regression analysis was computed to identify factors that affected the intention to leave. In the final model, variables with a P-value < 0.05 and adjusted odds ratio (AOR) with 95% confidence interval (CI) were used to declare the associated factors after model fitness was checked using the Hosmer–Lemeshow goodness-of-fit test (P = 0.25).

### Results

#### Socio-demographic characteristics of respondents

A total of 336 laboratory professionals answered the questionnaire with a response rate of 91.8%. The median age of the study participants was 27 with inter quartile range (IQR) of (26, 30) years. The majority 216 (64.3%) of the respondents were male, more than half 182 (54.2%) of whom were unmarried. Fifty-six percent of the respondents were B.Sc. degree graduates in laboratory technology and 53.9% had 1–5 years of work experience, 49% of the respondents worked in primary hospitals, and their median monthly salary was Ethiopian Birr (ETB) 3145 (IQR: 2514, 4725) (Table 1).

**Table 1:** Socio-demographic characteristics of laboratory professionals working in Amhara National Regional State public hospitals, 2016 (n = 336)

Variables	Category	Frequency (n)	Percentage (%)
Age in years	20–29	226	67.3
	30–39	99	29.5
	≥ 40	11	3.3
Sex	Male	216	64.3
	Female	120	35.7
Educational level	Diploma	128	38.0
	B.Sc. degree	188	56.0
	Above degree	20	6.0
Type of hospital	Referral	129	38.4
	General	42	12.5
	Primary	16	49.1
Work experience in years	< 1	31	9.2
	1–5	122	36.3
	6–10	142	42.3
	> 10	41	12.2
Marital status	Unmarried	182	54.2
	Married	154	45.8
Current position	Head	19	5.6
	Quality officer	18	5.4
	Safety officer	13	3.9
	Laboratory member	286	85.1
Monthly salary (ETB)	< 3145	129	38.5
	3145–3911	109	32.4
	3912–4725	32	9.5
	> 4725	66	19.6

*ETB* Ethiopian Birr

#### Intention to leave, organizational commitment and job satisfaction

The overall intention to leave hospitals among laboratory professionals in the study was 65.5% (95% CI 60–70). The median intention to leave the profession was 75% (IQR: 55, 85), whereas 179 (53.3%) of the LPs had intentions to leave their jobs.

Two hundred eighty-five (84.8%) of the LPs had a high level of satisfaction with co-worker relationships. On the other hand, 275 (81.8%), 247 (73.5%), 253 (75.3%) and 222 (66.1%) were reported to have been dissatisfied with payments and benefits, educational opportunities, recognition and rewards and working environments, respectively. Regarding organizational commitment, the majority (73.8%) of the respondents had a low level of continuance commitment (Table 2).

**Table 2:** Level of job satisfaction by different dimensions among laboratory professionals working in Amhara National Regional State public hospitals, Ethiopia, 2016 (n = 336)

Variables	Category	Frequency (n)	Percentage (%)
Benefits and pay	Satisfied	61	18.2
	Dissatisfied	275	81.8
Supervisor support	Satisfied	157	46.7
	Dissatisfied	179	53.3
Policy and strategy	Satisfied	85	25.3
	Dissatisfied	251	74.7
Coworker relationship	Satisfied	285	84.8
	Dissatisfied	51	15.2
Educational opportunity	Satisfied	89	26.5
	Dissatisfied	247	73.5
Nature of the work	Satisfied	129	38.4
	Dissatisfied	207	61.6
Responsibility	Satisfied	176	52.4
	Dissatisfied	160	47.6
Autonomy	Satisfied	170	50.6
	Dissatisfied	166	49.4
Workload	Low	164	48.8
	High	172	51.2
Performance appraisal	Satisfied	96	28.6
	Dissatisfied	240	71.4
Recognition	Satisfied	83	24.7
	Dissatisfied	253	75.3
Working environment	Satisfied	114	33.9
	Dissatisfied	222	66.1
Affective commitment	High	171	50.9
	Low	165	49.1
Normative commitment	High	101	30.1
	Low	235	69.9
Continuance commitment	High	88	26.2
	Low	248	73.8

#### Factors associated with intentions to leave

In the multivariable logistic regression analysis, eight variables were statistically significant. Accordingly, laboratory professionals who were dissatisfied with payments and benefits were 3.42 times more likely to leave the organizations than those who were satisfied (AOR: 3.42, 95% CI 1.39–8.42). Those who were dissatisfied with professional opportunities were 3.59 times more likely to leave their current working organizations than the satisfied LPs (AOR: 3.59, 95% CI 1.61–7.99). Similarly, respondents dissatisfied with recognition (AOR: 2.69, 95% CI 1.35–5.38) and working environments (AOR: 2.77, 95% CI 1.45–5.30) were more likely to leave their organizations than their counterparts. Moreover, those who had a high workload were 1.95 times more intended to leave their organizations than their counterparts (AOR: 1.95, 95% CI 1.06–3.57). Unmarried laboratory professionals were 2.46 times more likely to leave their organizations than those who were married (AOR: 2.46, 95% CI 1.32–4.58) (Table 3).

**Table 3:** Multivariable logistic regression analysis of factors associated with intention to leave among laboratory professionals working in Amhara National Regional State public hospitals, 2016 (n = 336)

Variables	Category	Intention to leave		COR (95% CI)	AOR (95% CI)
		Yes n (%)	No n (%)		
Pay and Benefits	Satisfied	12 (19.7)	49 (80.3)	1	1
	Dissatisfied	208 (75.6)	67 (24.4)	12.67 (6.36–25.24)***	3.89 (1.53–9.89)**
Supervisor support	Satisfied	94 (59.9)	63 (40.1)	1	1
	Dissatisfied	126 (70.4)	53 (29.6)	1.59 (1.01–2.50)*	1.52 (0.80–2.88)
Policy and strategy	Satisfied	49 (57.6)	36 (42.4)	1	1
	Dissatisfied	171 (68.1)	80 (31.9)	1.59 (1.01–2.50)*	0.62 (0.27–1.39)
Educational opportunity	Satisfied	22 (24.7)	67 (75.3)	1	1
	Dissatisfied	198 (80.2)	49 (19.8)	12.30 (6.93–21.85)***	3.59 (1.61–7.99)**
Nature of the work	Satisfied	77 (59.7)	52 (40.3)	1	1
	Dissatisfied	143 (69.1)	64 (30.9)	1.50 (0.95–2.38)*	0.72 (0.34–1.49)
Workload	Low	90 (54.9)	74 (45.1)	1	1
	High	130 (75.6)	42 (24.4)	2.54 (1.60–4.04)***	1.94 (1.04–3.63)*
Performance appraisal	Satisfied	54 (56.2)	42 (43.8)	1	1
	Dissatisfied	166 (69.2)	74 (30.8)	1.74 (1.07–2.84)*	0.78 (0.35–1.72)
Recognition	Satisfied	36 (43.4)	47 (56.6)	1	1
	Dissatisfied	184 (72.7)	69 (27.3)	3.48 (2.08–5.82)***	2.69 (1.35–5.38)**
Work environment	Satisfied	42 (36.8)	72 (63.2)	1	1
	Dissatisfied	178 (80.2)	44 (19.8)	6.93 (4.19–11.47)***	2.77 (1.45–5.30)**
Affective commitment	High	89 (52.0)	82 (48.0)	1	1
	Low	131 (79.4)	34 (20.6)	3.55 (2.19–5.74)***	2.05 (1.10–3.82)*
Marital status	Married	77 (50.0)	77 (50.0)	1	
	Unmarried	143 (78.6)	39 (21.4)	3.66 (2.28–5.89)***	2.46 (1.32–4.58)**
Educational level	Diploma	77 (60.2)	51 (39.8)	1	
	Degree	126 (67.0)	62 (33.0)	1.34 (0.84–2.14)	9.09 (0.45–183.49)
	Above degree	17 (85.0)	3 (15.0)	3.75 (1.04–13.46)	11.86 (0.36–383.59)
Level of hospital	Primary	109 (66.1)	56 (33.9)	1	
	General	22 (52.4)	20 (47.6)	0.56 (0.28–1.12)*	0.36 (0.14–0.89)*
	Referral	89 (69.0)	40 (31.0)	1.14 (0.69–1.87)	0.89 (0.44–1.81)
Monthly salary (ETB)	< 3145	78 (60.5)	51 (39.5)	1	1
	3145–3911	72 (66.1)	37 (33.9)	1.27 (0.74–2.16)	0.79 (0.37–1.64)
	3912–4725	18 (56.2)	14 (43.8)	0.84 (0.38–1.83)	0.37 (0.13–1.03)
	> 4725	52 (78.8)	14 (21.2)	2.42 (1.22–4.80)	2.55 (0.97–6.66)

*AOR* adjusted odds ratio, *CI* confidence interval, *COR* crude odds ratio, *ETB* Ethiopian Birr

* P < 0.05, ** P < 0.01, *** P < 0.001, 1: reference category

### Discussion

The findings showed that 65.5% of the respondents had intentions to leave the hospitals. This result is higher than those of studies conducted at the University of Gondar referral hospital on health professionals, 52.5% [17] and nurses working at governmental healthcare institutions of East Gojjam Zone, 59.4% [21]. Additionally, our finding is much higher than those of studies conducted among health workers in Tanzania (18.8%), Malawi (26.5%), and South Africa 41.4% [15]. These differences could be due to variations in health institution infrastructures, study settings and participants regard to nurses alone.

However, it is lower than the result of studies done among health professionals at Sidama zone public health facilities (84.3%) as well as Yirgalem and Hawassa referral hospitals (83.7%) [15, 22]. This discrepancy could have resulted from differences in the infrastructures of the health institutions, study areas, and study participants that might have affected intentions to leave.

Our finding shows that LPs dissatisfied with payments and benefits were 3.89 more intended to leave their hospital compared to their counterparts. This finding is consistent with the result of other similar studies conducted in Ethiopia [19, 21]. This could be explained by the disproportionality of tasks assigned and benefits given which pushed professionals to search for new jobs, while satisfied professionals wanted to remain within organizations because of needs to maintain benefits.

Our finding also showed that LPs who were dissatisfied with educational opportunities were 3.59 times more likely to leave their organizations compared with their satisfied counterparts. Poor training opportunities also increased intentions to leave as reported by other studies [19, 21, 23]. This can be explained by the fact that less professional opportunities may increase job dissatisfaction because of the absence of chances to grow and develop own skills and abilities.

Intentions to leave were higher among respondents who were dissatisfied with the recognition and reward granted compared to their counterparts. This might be because satisfied professionals believe that leaving organizations that reward would be costly in that the opportunities might be unlikely to obtain elsewhere. This finding is supported by those of studies done on Jordanian nurses and revealed the direct and buffering effects of recognition to the performance of nurses on the intention to stay on jobs [24] (see Herzberg Two Factor Theory of Motivation [25].

Our finding also shows that LPs who were dissatisfied with working environments were three times more likely to leave their hospitals compared to their counterparts. This finding is in agreement with those of studies done in Sidama and Jimma zones public health facilities [15, 26] and also supported by Herzberg Two Factor Theory of Motivation which identifies recognition, work conditions, the nature of work and responsibility as factors that influence employee intentions to stay or leave by affecting their satisfaction needs [25]. The other possible explanation could be that substandard working conditions or lack of important facilities in workplaces such as proper lighting, furniture, restrooms, and other health and safety provisions reduce the convenience of employees and hinder their intentions to stay on jobs.

Our study identified that LPs with high workload were more likely to leave their organizations was congruent with the finding of other studies [26, 27]. This could be because excess load increases pressure and results in high fatigue which may lead employees to seek jobs elsewhere.

Moreover, the findings show that LPs with low affective commitment were two times more likely to intend to leave their organizations compared to those with high affective commitment. This finding is supported by other studies in which committed employees are likely to remain with their organizations [28, 29]. That is because if employees have a sense of belonging or are involved and linked emotionally, they want to stay within the organization.

### Conclusion and recommendations

Laboratory professional’s intention to leave public hospitals in ANRS was found to be high and might be detrimental to both organizations and employees. Dissatisfaction with training opportunities, payments and benefits, recognition at work, working environments, low affective commitment, and high workload were the factors that influence intentions to leave. Policymakers and hospital administrators need to develop and institutionalize evidence-based retention strategies to reduce LPs’ intention to leave.

## Limitations of the study

The use of self-reporting measures may have some potential of minimizing reporting bias, which may occur due to respondents’ interpretations of questions. Furthermore, the study was not triangulated with a qualitative method. The other limitation was lack of follow-up that could enable the researcher to compare participant intentions to leave or stay with their actual turnover actions.

## Data Availability

Data will be available upon reasonable request from the corresponding author.

## Abbreviations

ANRS: Amhara National Regional State
AOR: adjusted odds ratio
CI: confidence interval
COR: crude odds ratio
ETB: Ethiopian Birr
IQR: inter quartile range
LP: laboratory professional
SPSS: Statistical Package for the Social Sciences
